# Supplementary material for: Projecting genetic associations through gene expression patterns highlights disease etiology and drug mechanisms
Source: Nat Commun. 2023 Sep 9;14:5562. doi: 10.1038/s41467-023-41057-4 (PMC10492839; doi:10.1038/s41467-023-41057-4)
Supplement: Supplementary file 1 — Supplementary Information [file 41467_2023_41057_MOESM1_ESM.pdf]

Supplementary information

Supplementary Note 1: mean type I error rates and calibration of LV-based regression model

We assessed our GLS model type I error rates (proportion of p-values below 0.05) and calibration using a null model of random traits and genotype data from 1000 Genomes Phase III. We selected 312 individuals with European ancestry, and then analyzed 1,000 traits drawn from a standard normal distribution $\mathcal{N}(0, 1)$. We ran all the standard procedures for the TWAS approaches (S-PrediXcan and S-MultiXcan), including: 1) a standard GWAS using linear regression under an additive genetic model, 2) different GWAS processing steps, including harmonization and imputation procedures as defined in[1], 3) S-PrediXcan and S-MultiXcan analyses. Below we provide details for each of these steps.

Step 1 - GWAS. We performed standard QC procedures such as filtering out variants with missing call rates eexceeding 0.01, MAF below 1% or MAC below 20, and HWE below 1e-6, and removing samples with high sex-discrepancy and high-relatedness (first and second degree). We included sex and the top 20 principal components as covariates, performing the association test on 5,923,554 variants across all 1,000 random phenotypes.

Step 2 - GWAS processing. These steps include harmonization of GWAS and imputation of z-scores, which are part of the TWAS pipeline and are needed in order to ensure an acceptable overlap with SNPs in prediction models. The scripts to run these steps are available in[2]. These procedures were run for all 1,000 random phenotypes and generated a total number of 8,325,729 variants, including those with original and imputed z-scores.

Step 3 - TWAS. We processed the imputed GWAS with S-PrediXcan using the MASHR prediction models on 49 tissues from GTEx v8. Then, S-MultiXcan was ran using the GWAS and S-PrediXcan outputs to generate gene-trait association p-values.

Finally, we ran our GLS model (Equation (7)) to compute an association between each of the 987 LVs in MultiPLIER and the 1,000 S-MultiXcan results on random phenotypes. For this, we built a gene correlation matrix specifically for this cohort (see Methods). Then, we compared the GLS results with an equivalent, baseline ordinarly least squares (OLS) model assuming independence between genes. Supplementary Figure 1 compares the distribution of p-values of the OLS and GLS models. The GLS model has a slightly smaller mean type I error rate (0.0558, SD=0.0127) than the baseline OLS model (0.0584, SD=0.0140), and p-values follow more closely the expected uniform distribution. Importantly, the GLS model is able to correct for LVs with adjacent and highly correlated genes at the top such as LV234 (Supplementary Figure 2), LV847 (Supplementary Figure 3), LV45 (Supplementary Figure 4), or LV800 (Supplementary Figure 5), among others. In contrast and as expected, the OLS model has higher mean type I errors and smaller-than-expected p-values in all these cases.

We also detected other LVs with higher-than-expected mean type I errors for both the GLS and OLS models, although they don't have a relatively large number of adjacent genes at the top. One example is LV914, shown in Supplementary Figure 6. Inflation in these LVs might be explained by inaccuracies in correlation estimates between the individual-level MultiXcan model and its summary-based version (see Methods). Therefore, we flagged those with a type I error rate larger than 0.07 (127 LVs) and excluded them from our main analyses. We didn't see signs of inflation when applying the method in real data (Supplementary Figure 7).

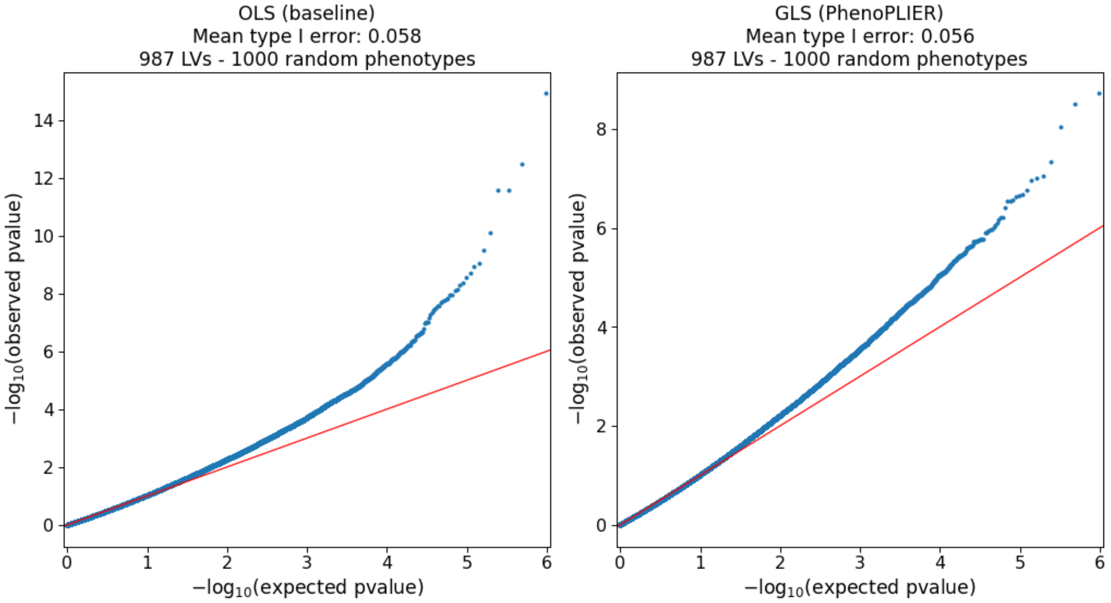

Supplementary Figure 1: **QQ-plots for OLS (baseline) and GLS (PhenoPLIER) models on random phenotypes.**

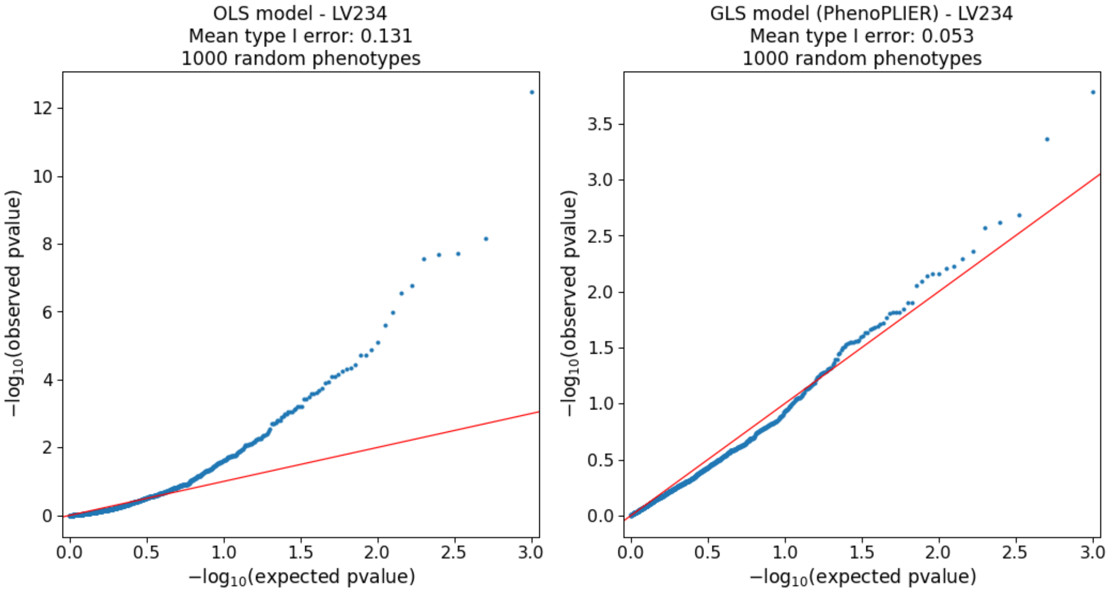

Supplementary Figure 2: **QQ-plots for LV234 on random phenotypes.** Among the top 1% of genes in this LV, 17 are located in band 6p22.2, 5 in 6p22.1 and 3 in 7q11.23.

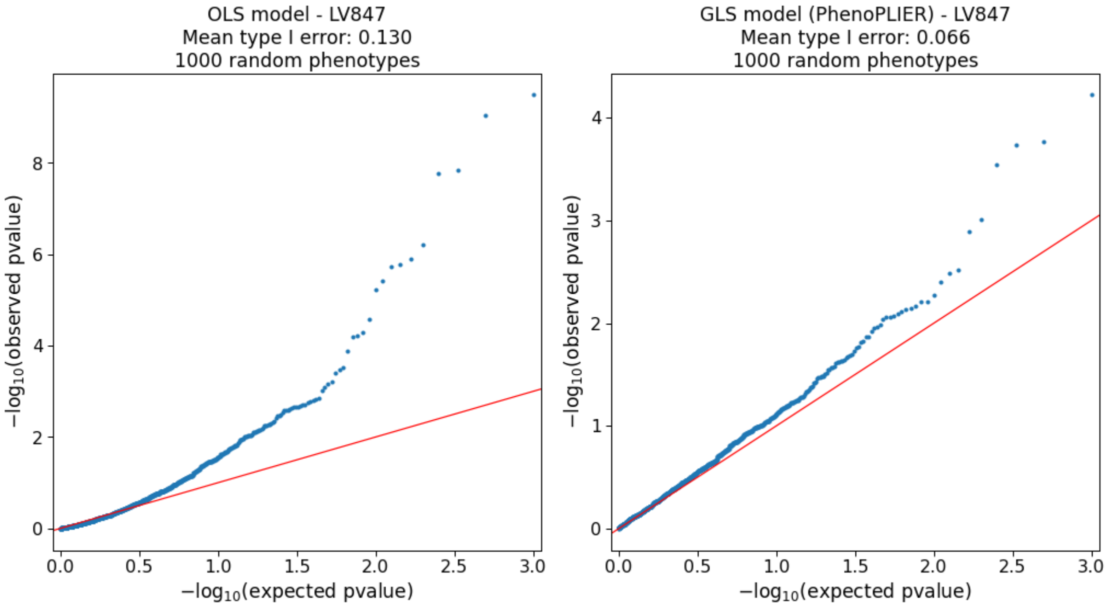

Supplementary Figure 3: **QQ-plots for LV847 on random phenotypes.** Among the top 1% of genes in this LV, 15 are located in band 6p22.2, 5 in 6p22.1 and 2 in 15q26.1.

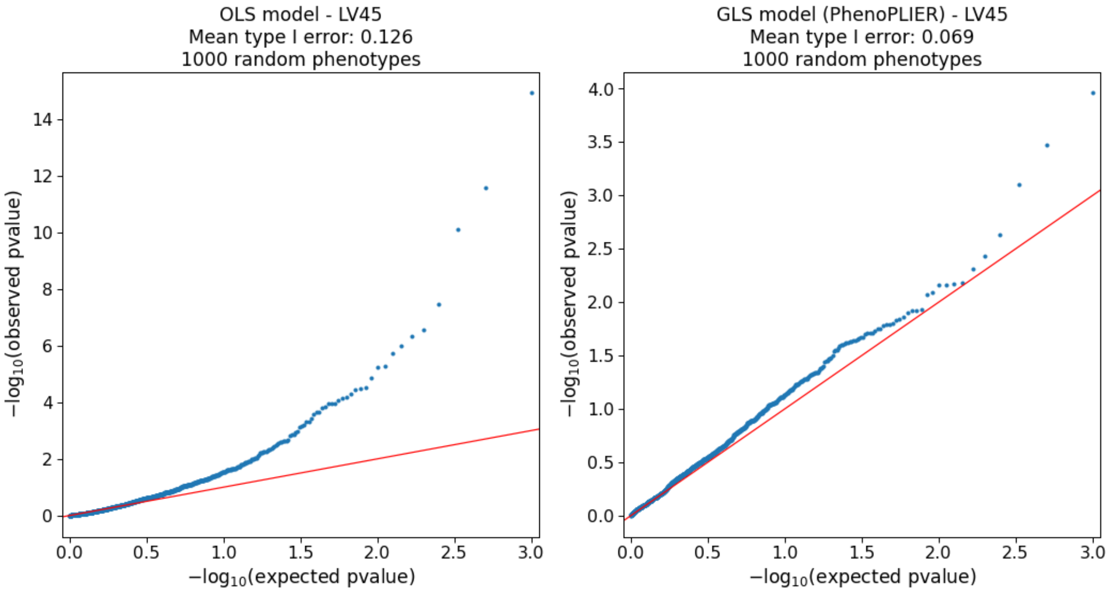

Supplementary Figure 4: **QQ-plots for LV45 on random phenotypes.** Among the top 1% of genes in this LV, 12 are located in band 6p22.2, 6 in 6p22.1 and 3 in 1q23.3.

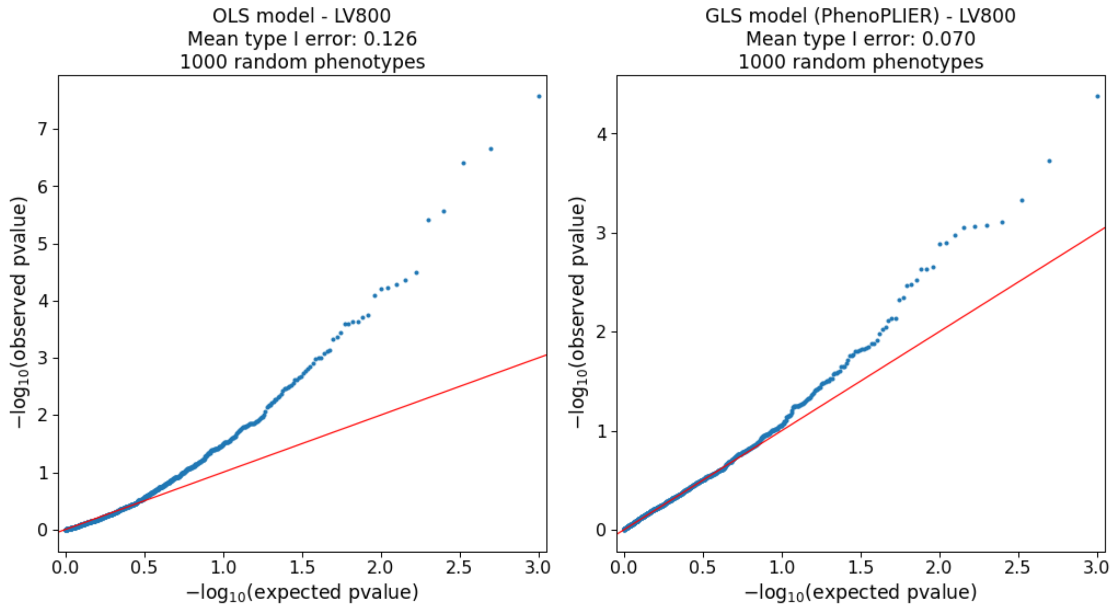

Supplementary Figure 5: **QQ-plots for LV800 on random phenotypes.** Among the top 1% of genes in this LV, 16 are located in band 19q13.43, 9 in 19p13.2 and 9 in 19q13.31.

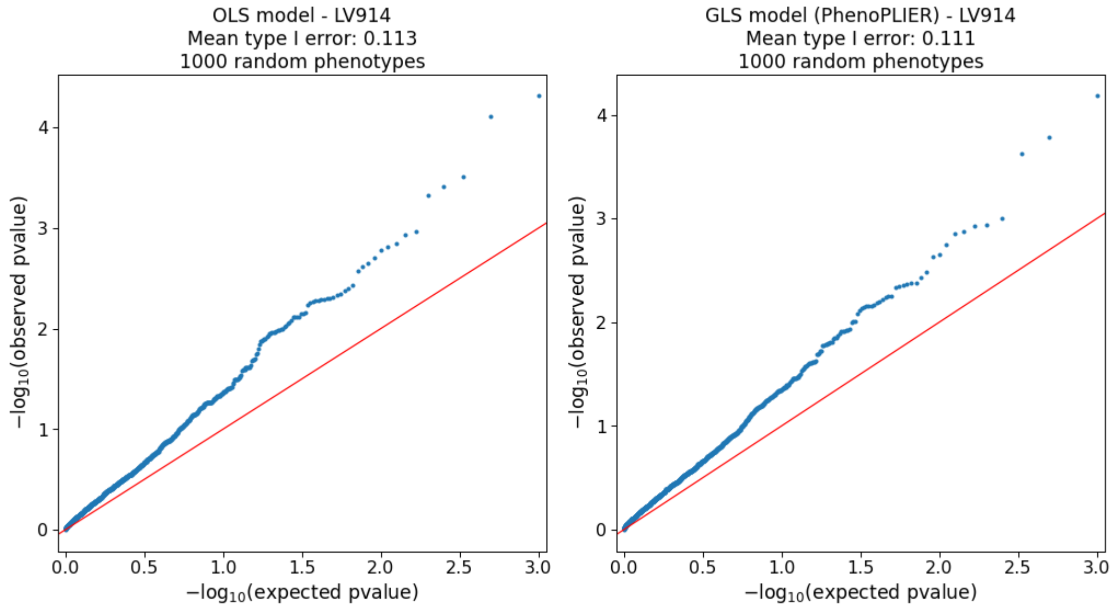

Supplementary Figure 6: **QQ-plots for LV914 on random phenotypes.** Among the top 1% of genes in this LV, 2 are located in band 13q13.3, 2 in 7p15.2 and 2 in 19q13.2.

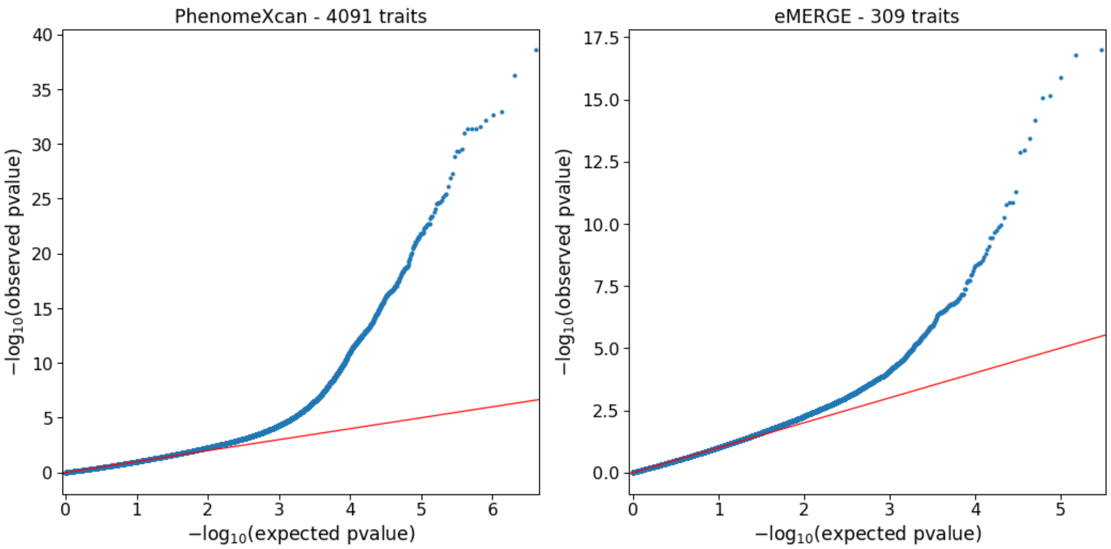

Supplementary Figure 7: **QQ-plots of LV-trait associations in real data.** QQ-plot in PhenomeXcan (left, discovery cohort) across 4,091 traits and 987 LVs, and eMERGE (right, replication cohort) across 309 traits and 987 LVs.

CRISPR-Cas9

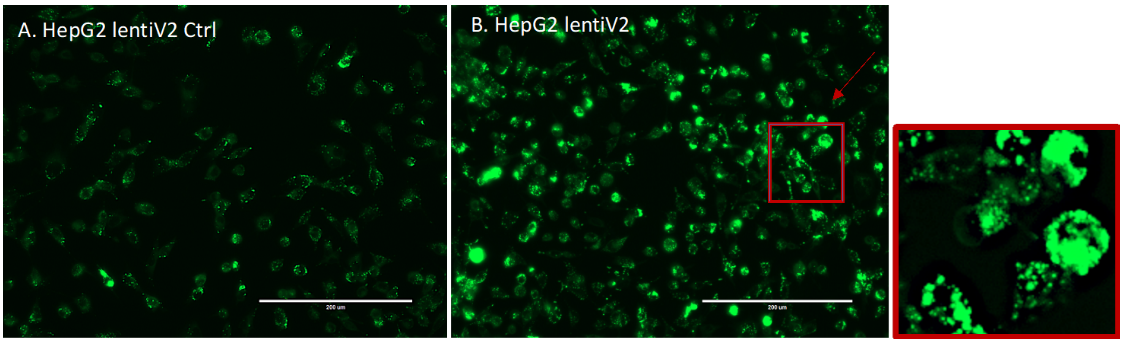

Supplementary Figure 8: **EVOS Fluorescence Microscope Image Capture.** A. HepG2_lentiV2_Ctrl with no-viral transduction. B. HepG2_lentiV2 with viral transduction. Both no-viral transduction Control (A) and lentiviral transduction (B) HepG2 cells were stained with LipidSpot™488. The CRISPR screening process was performed once, but we conducted two selections (high and low fluorescence) with a control of no/before selection. Subsequently, we generated three technical replicates for the DNA-seq libraries under each condition. In order to mitigate false positives resulting from the single-screen process, we overlapped the candidates from multiple pairwise differential analyses and selected the genes that were consistent between selections.

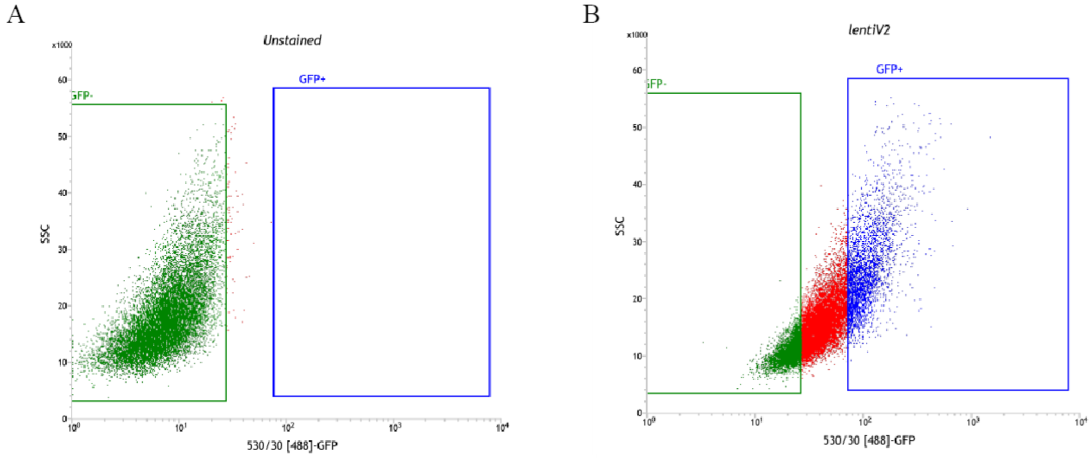

Statistics: *Unstained*

Populations	Events	% Total	% Parent
All Events	20,000	100.00%	####
Singlets	19,003	95.02%	95.02%
Cells	13,526	67.63%	71.18%
GFP-	13,453	67.26%	99.46%
GFP+	0	0.00%	0.00%

Statistics: *lentiV2*

Populations	Events	% Total	% Parent
All Events	20,000	100.00%	####
Singlets	19,692	98.46%	98.46%
Cells	17,227	86.14%	87.48%
GFP-	3,738	18.69%	21.70%
GFP+	3,418	17.09%	19.84%

Supplementary Figure 9: **Fluorescence-Activated Cell Sorting Gate Setting.** A. HepG2_UnStained WT. B. HepG2_lentiV2 with viral transduction stained with LipidSpot™488. HepG2_lentiV2 cells were FAC sorted, 20% of GFP-High and 20% of GFP-Low cell populations were collected.

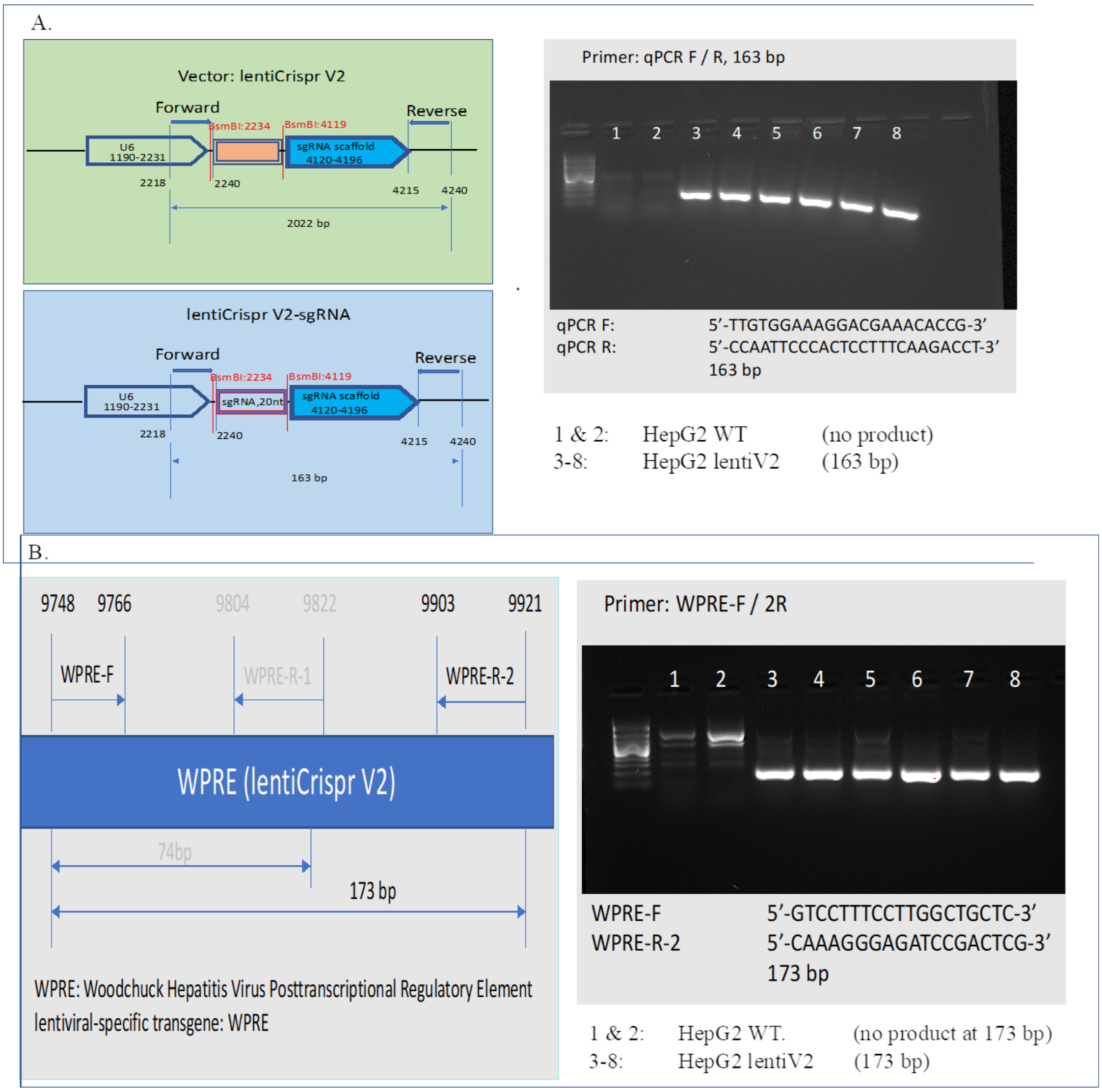

Supplementary Figure 10: **Verification of sgRNA cassette and lentiV2 transgene.** A. 20nt sgRNA cassette was verified in lentiV2 transduced genomic DNA population, 163 bp PCR product obtained, while WT HepG2 didn't possess the cassette, thus, no PCR product. B. lentiviral-specific transgene WPRE was verified in lentiV2 transduced genomic DNA population, while no transduced WT didn't have the transgene, therefore, no 173 bp PCR product observed. For both panels A and B, 100 bp ladder was used in Lane 0. The CRISPR screening process was performed once, but we conducted two selections (high and low fluorescence) with a control of no/before selection. Subsequently, we generated three technical replicates for the DNA-seq libraries under each condition. In order to mitigate false positives resulting from the single-screen process, we overlapped the candidates from multiple pairwise differential analyses and selected the genes that were consistent between selections.

Primers for Generating Illumina Libraries

P5/P7 flowcell attachment sequence
Illumina sequencing primer
Vector primer binding sequence
Stagger region / Barcode region

P5 primer :
5'AATGATACGGCGACCACCGAGATCTACACTCTTTCCCTACACGACGCTCTTCCGATCT[s]TTGTGGAAAGGACGAAACACCG

P7 primer:
5'CAAGCAGAAGACGGCATACGAGATNNNNNNNNGTGACTGGAGTTCAGACGTGTGCTCTTCCGATCTCCAATTCCCACTCCTTTCAAGACCT

P5 Stagger Region sequences ([s])	
	Stagger Sequence to include in P5 primer, 5'-3'
P5 0 nt	
P5 1nt	C
P5 2 nt	GC
P5 3 nt	AGC
P5 4 nt	CAAC
P5 6 nt	TGCACC
P5 7nt	ACGCAAC
P5 8nt	GAAGACCC

P7 barcode (index) sequences (NNNNNNNN)	
	Sequence to include in P7 primer, 5'-3'
P7-001	CGGTTCAA
P7-002	GCTGGATT
P7-003	TAACTCGG
P7-004	TAACAGTT
P7-005	ATACTCAA
P7-006	GCTGAGAA
P7-007	ATTGGAGG
P7-008	TAGTCTAA
P7-009	CGGTGACC

Supplementary Figure 11: **Primers for generating illumina libraries.** Sequences are provided in a readable format in Supplementary Data 8.

A. Construct For Illumina Library Generation

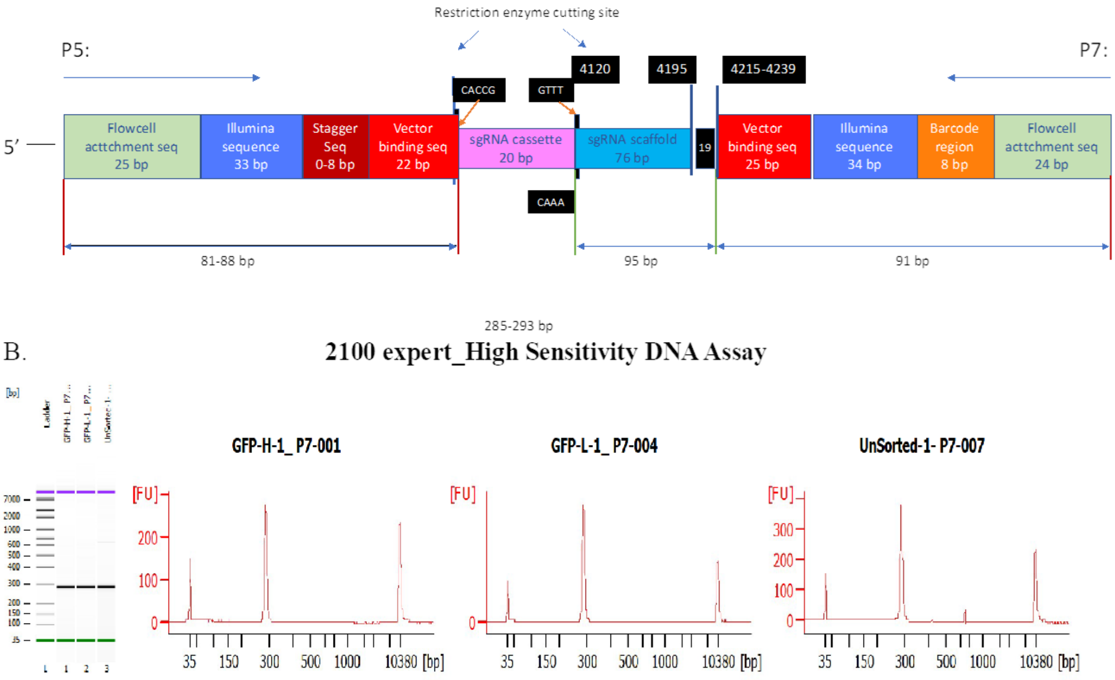

Supplementary Figure 12: **Illumina library generation.** A. Construct for generating Illumina libraries. B. Final Illumina library from HS DNA —showed a single ~285bp peak was generated. The CRISPR screening process was performed once, but we conducted two selections (high and low fluorescence) with a control of no/before selection. Subsequently, we generated three technical replicates for the DNA-seq libraries under each condition. In order to mitigate false positives resulting from the single-screen process, we overlapped the candidates from multiple pairwise differential analyses and selected the genes that were consistent between selections.

Gene modules enrichment for lipids gene-sets

Supplementary Table 1: Gene modules (LVs) nominally enriched (using FGSEA[3]) for the lipids-increasing gene-set from the CRISPR-screen (unadjusted p-values < 0.01). LVs significantly aligned with pathways (FDR < 0.05) from the MultiPLIER models are shown in boldface.

Gene module	Lipids gene-set	Leading edge	p-value
**LV246**	increase	*DGAT2*, *ACACA*	0.0035
LV702	increase	*ACACA*, *DGAT2*	0.0046
**LV607**	increase	*ACACA*, *DGAT2*	0.0058
LV890	increase	*ACACA*, *DGAT2*	0.0067
**LV74**	increase	*MBTPS1*, *DGAT2*	0.0078
**LV865**	increase	*ACACA*, *DGAT2*	0.0092
LV841	increase	*ACACA*, *DGAT2*	0.0096

Supplementary Table 2: Gene modules (LVs) nominally enriched (using FGSEA[3]) for the lipids-decreasing gene-set from the CRISPR-screen (unadjusted p-values < 0.01). LVs significantly aligned with pathways (FDR < 0.05) from the MultiPLIER models are shown in boldface.

Gene module	Lipids gene-set	Leading edge	p-value
LV520	decrease	*FBXW7*, *TCF7L2*	0.0006
LV801	decrease	*UBE2J2*, *TCF7L2*	0.0022
LV512	decrease	*FBXW7*, *TCF7L2*	0.0025
**LV612**	decrease	*PTEN*, *FBXW7*	0.0036
LV41	decrease	*PCYT2*, *TCF7L2*	0.0041
**LV838**	decrease	*UBE2J2*, *TCF7L2*	0.0070
LV302	decrease	*TCF7L2*, *PTEN*	0.0083
LV959	decrease	*TCF7L2*, *PTEN*	0.0092

Supplementary Note 2: Cluster analyses under the null hypothesis of no structure in the data

For our clustering pipeline, we simulated different escenarios where there is no structure in the input data matrix $\hat{\mathbf{M}}$ (gene-trait associations from PhenomeXcan projected into the latent gene expression representation). For this, we simulated two cases where any groupings of traits are removed: 1) the gene-trait association matrix $\mathbf{M}$ (from S-MultiXcan) does not have any meaningful structure to find groups of traits, while preserving the latent variables in $\mathbf{Z}$ from the MultiPLIER models; and 2) the latent variables in matrix $\mathbf{Z}$ does not have any meaningful structure to find groups of traits, while preserving the gene-trait association matrix $\mathbf{M}$.

For the first scenario, we shuffled genes in $\mathbf{M}$ for each trait, and this randomized matrix was then projected into the latent space. For the second scenario, we projected matrix $\mathbf{M}$ into the latent space, and then shuffled LVs in $\hat{\mathbf{M}}$ for each trait. For each of these scenarios, we ran exactly the same clustering pipeline we used for the real data (Methods), generating an ensemble of partitions that was later combined using the same consensus functions to derive the final partitions of traits. Finally, we computed 1) stability statistics on the ensemble partitions from different algorithms and 2) the agreement of the final consensus partition with the ensemble.

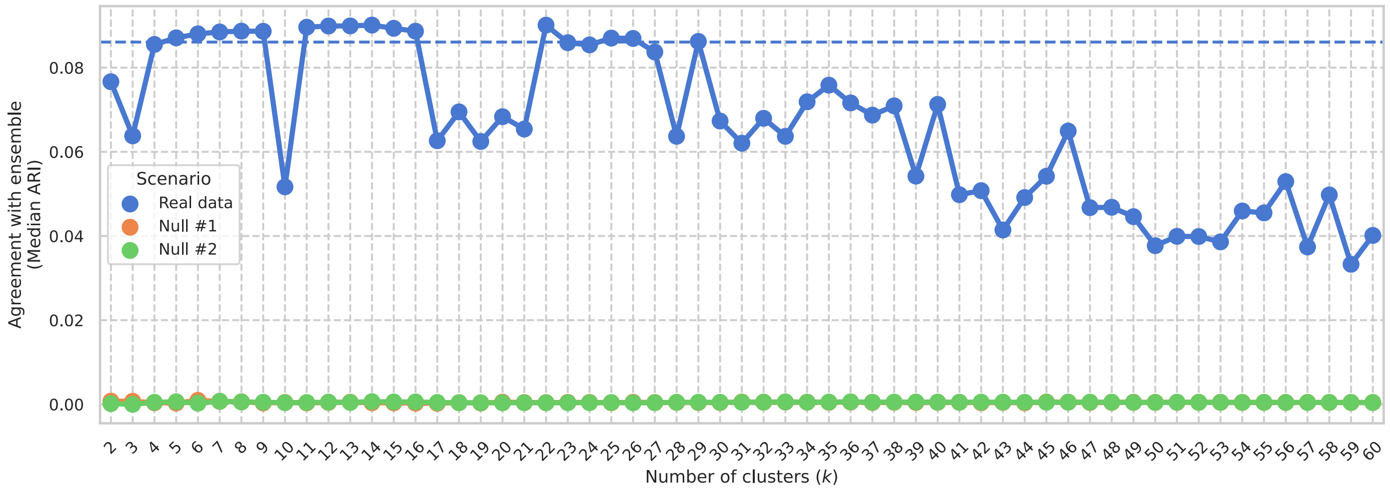

Supplementary Figure 13: **Agreement of consensus partitions with ensemble.** A real and two simulated scenarios with no data structure are shown. For each scenario, one final consensus partition was derived for each k from 2 to 60 (x-axis) following our clustering pipeline. For each partition, the agreement with the corresponding ensemble was computed using the ARI (y-axis). For the real data scenario, partitions with an agreement above the 75th percentile (dashed line) were selected for follow-up analyses in the main text.

The results of this analysis (Supplementary Figure 13) show that, under the two simulated null scenarios, the agreement of the consensus partitions with the ensemble is very close to zero. This means, as expected, that there is no consensus among ensemble partitions generated with different clustering algorithms and data representations. In contrast, using the real data, the consensus clustering approach finds trait pairs that are grouped together across the different members of the ensemble. The partitions above the 75th percentile were considered in the main analyses, and are shown in the clustering tree in Figure 6.

Cluster-specific and general transcriptional processes associated with disease

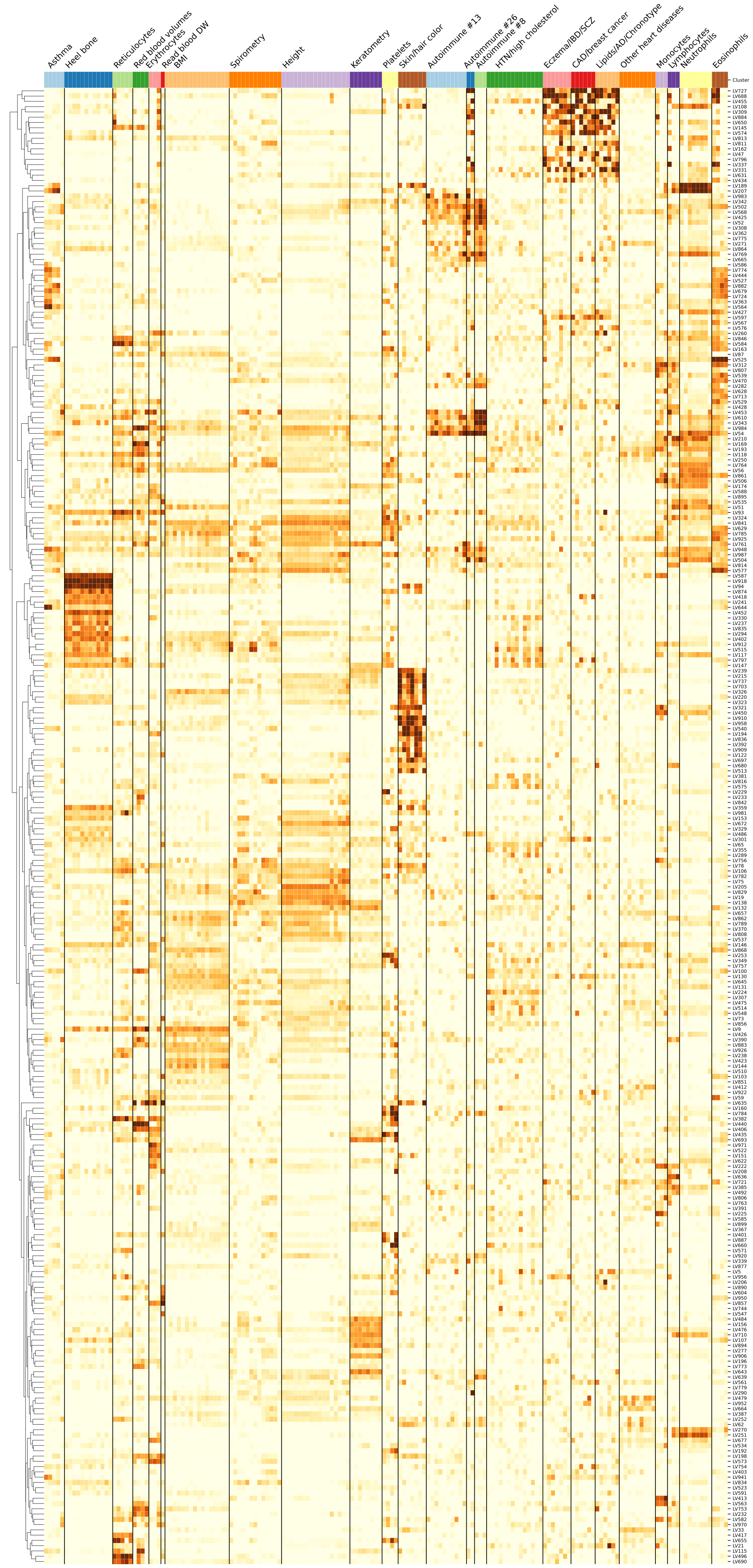

Supplementary Figure 14: **Cluster-specific and general transcriptional processes associated with disease using novel LVs.** The plot shows a submatrix of $\hat{\mathbf{M}}$ for the main trait clusters at k=29, considering only LVs (rows) that are not aligned with any pathway. Standardized values from -6 (lighter color) to 21 (darker color).

Latent variables (gene modules) information

LV603

Supplementary Table 3: Pathways aligned to LV603 from the MultiPLIER models.

Pathway	AUC	FDR
IRIS Neutrophil-Resting	0.91	4.51e-35
SVM Neutrophils	0.98	1.43e-09
PID IL8CXCR2 PATHWAY	0.81	7.04e-03
SIG PIP3 SIGNALING IN B LYMPHOCYTES	0.77	1.95e-02

Supplementary Table 4: Significant trait associations of LV603 in PhenomeXcan.

Trait description	Sample size	Cases	FDR
Basophill percentage	349,861		1.19e-10
Basophill count	349,856		1.89e-05
Treatment/medication code: ispaghula husk	361,141	327	1.36e-02

Supplementary Table 5: Significant trait associations of LV603 in eMERGE.

Phecode	Trait description	Sample size	Cases	FDR
No significant associations				

LV246

Supplementary Table 6: Pathways aligned to LV246 from the MultiPLIER models.

Pathway	AUC	FDR
REACTOME FATTY ACID TRIACYLGLYCEROL AND KETONE BODY METABOLISM	0.89	3.97e-16
REACTOME METABOLISM OF LIPIDS AND LIPOPROTEINS	0.67	1.14e-08
REACTOME TRIGLYCERIDE BIOSYNTHESIS	0.86	6.52e-04
KEGG PYRUVATE METABOLISM	0.82	2.66e-03
KEGG PROPANOATE METABOLISM	0.83	4.27e-03

Supplementary Table 7: Significant trait associations of LV246 in PhenomeXcan.

Trait description	Sample size	Cases	FDR
Triglycerides NMR	21,559		1.66e-26
LDL Cholesterol NMR	13,527		3.92e-26
High cholesterol (self-reported)	361,141	43,957	1.08e-24
Cholesterol lowering medication	193,148	24,247	4.28e-24
Treatment/medication code: simvastatin	361,141	40,921	2.56e-19
CH2DB NMR	24,154		1.05e-15
Cholesterol lowering medication	165,340	38,057	9.58e-15
Treatment/medication code: atorvastatin	361,141	10,805	2.54e-14
Illnesses of mother: Alzheimer's disease/dementia	331,041	28,507	2.76e-08
Illnesses of father: Alzheimer's disease/dementia	312,666	15,022	2.76e-08
Alzheimers Disease	54,162	17,008	1.10e-07
Non-butter spread type details: Flora Pro-Active or Benecol	190,094	29,048	5.63e-07
Illnesses of siblings: Alzheimer's disease/dementia	279,062	1,609	6.16e-07
Any dementia	361,194	243	2.86e-05
Illnesses of father: None of the above (group 1)	314,797	116,736	3.56e-05
Medication for cholesterol, blood pressure, diabetes, or take exogenous hormones (females)	193,148	133,338	1.10e-04
Treatment/medication code: lipitor 10mg tablet	361,141	2,584	1.55e-04
Treatment/medication code: rosuvastatin	361,141	2,227	1.37e-03
Illnesses of father: Heart disease	318,570	104,110	1.89e-03
Dementia	361,194	157	9.58e-03
Mother still alive	355,029	140,246	1.76e-02
Job SOC coding: Librarians	91,149	1,248	3.22e-02
Alzheimer's disease	361,194	119	3.61e-02

Supplementary Table 8: Significant trait associations of LV246 in eMERGE.

Phecode	Trait description	Sample size	Cases	FDR
272.11	Hypercholesterolemia	40,786	14,138	4.40e-09
272.1	Hyperlipidemia	55,843	29,195	3.57e-07
272	Disorders of lipoid metabolism	55,892	29,244	3.79e-07
292.3	Memory loss	48,785	2,094	1.80e-02

LV116

Supplementary Table 9: Pathways aligned to LV116 from the MultiPLIER models.

Pathway	AUC	FDR
REACTOME INTERFERON SIGNALING	0.84	3.48e-09
SVM Macrophages M1	0.92	2.09e-05
REACTOME INTERFERON ALPHA BETA SIGNALING	0.94	3.36e-05
REACTOME CYTOKINE SIGNALING IN IMMUNE SYSTEM	0.67	1.53e-04
IRIS DendriticCell-LPSstimulated	0.65	1.09e-03
KEGG CYTOSOLIC DNA SENSING PATHWAY	0.84	3.22e-03
REACTOME NEGATIVE REGULATORS OF RIG I MDA5 SIGNALING	0.81	1.61e-02

LV931

Supplementary Table 10: Pathways aligned to LV931 from the MultiPLIER models.

Pathway	AUC	FDR
MIPS SPLICEOSOME	0.63	3.13e-02
PID TGFBRPATHWAY	0.71	3.99e-02

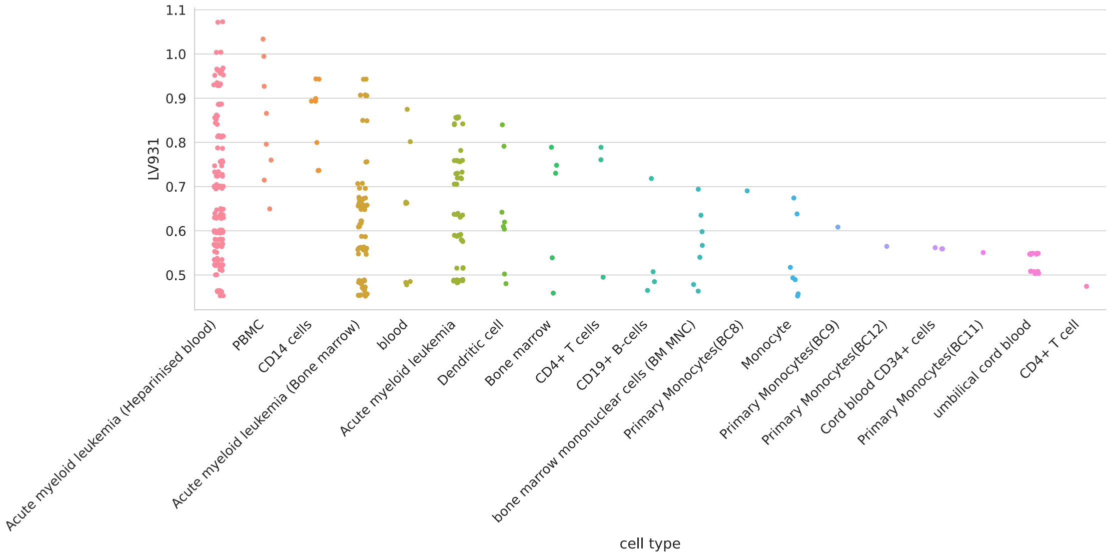

Supplementary Figure 15: **Cell types for LV931.**

LV66

Supplementary Table 11: Pathways aligned to LV66 from the MultiPLIER models.

Pathway	AUC	FDR
REACTOME METABOLISM OF LIPIDS AND LIPOPROTEINS	0.62	3.12e-04

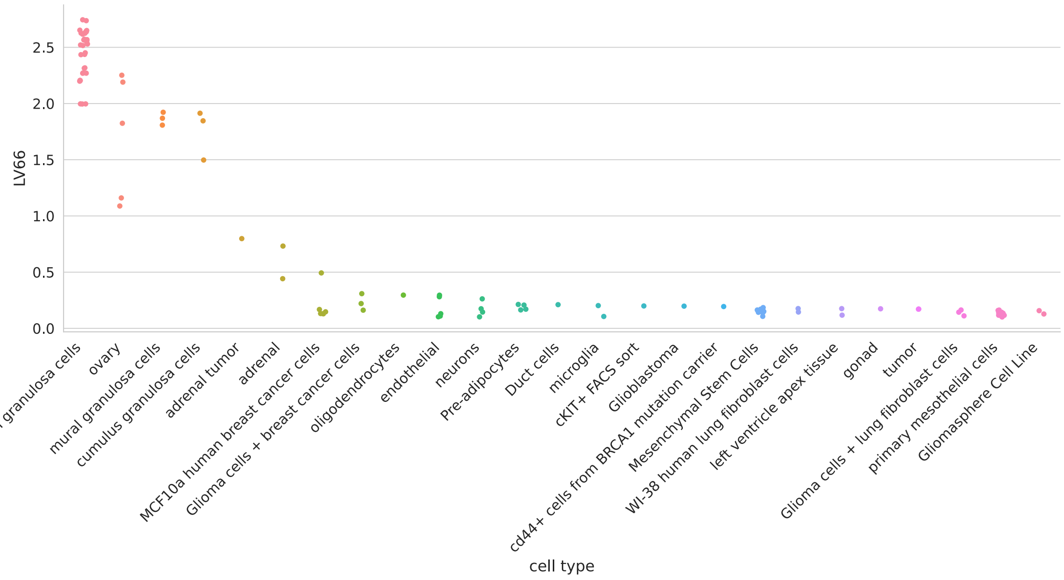

Supplementary Figure 16: **Cell types for LV66.**

LV928

Supplementary Table 12: Pathways aligned to LV928 from the MultiPLIER models.

Pathway	AUC	FDR
DMAP ERY3	0.81	1.16e-24
DMAP ERY4	0.78	2.49e-17

Supplementary Table 13: Significant trait associations of LV928 in PhenomeXcan.

Trait description	Sample size	Cases	FDR
Mean sphered cell volume	344,729		1.60e-20
Mean corpuscular haemoglobin concentration	350,468		1.42e-17
Mean reticulocyte volume	344,728		1.77e-17
Reticulocyte count	344,729		2.28e-10
Reticulocyte percentage	344,728		1.37e-09
Red blood cell (erythrocyte) distribution width	350,473		2.90e-09
Reticulocyte Count	173,480		1.09e-07
Mean corpuscular volume	350,473		1.46e-03
High light scatter reticulocyte count	344,729		3.49e-03
Age at first episode of depression	61,033		1.33e-02
High Light Scatter Reticulocyte Count	173,480		1.48e-02
Mean corpuscular haemoglobin	350,472		4.02e-02

Supplementary Table 14: Significant trait associations of LV928 in eMERGE.

Phecode	Trait description	Sample size	Cases	FDR
No significant associations				

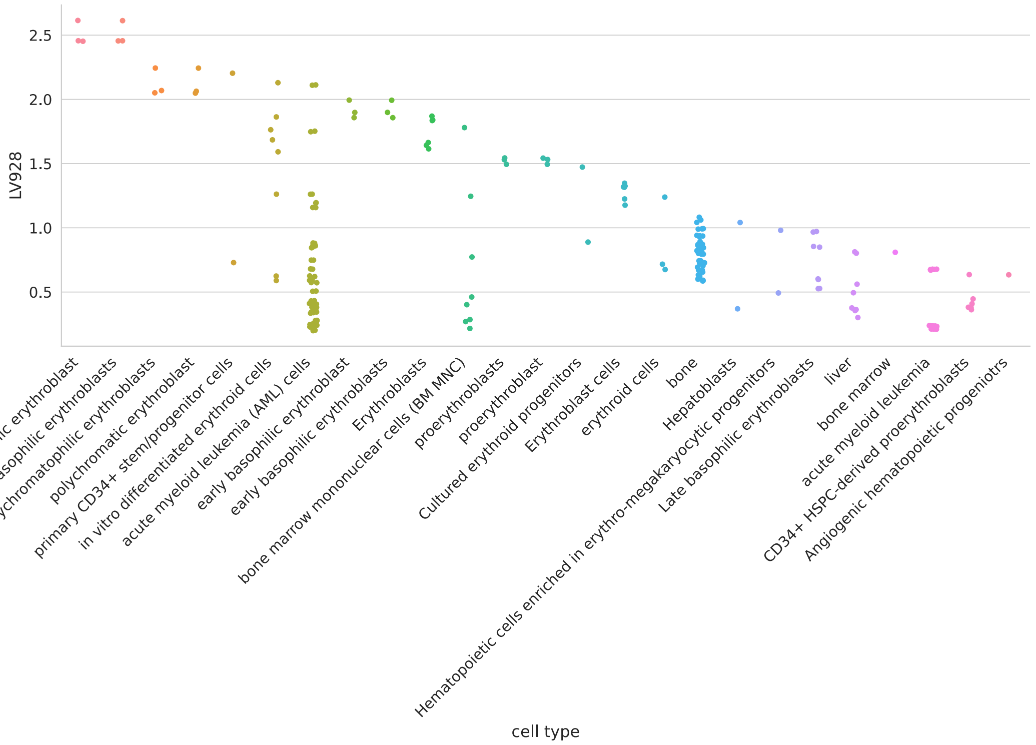

Supplementary Figure 17: **Cell types for LV928.**

LV30

Supplementary Table 15: Pathways aligned to LV30 from the MultiPLIER models.

Pathway	AUC	FDR
DMAP ERY3	0.95	5.62e-52
DMAP ERY4	0.98	5.28e-51
DMAP ERY5	0.98	1.96e-49

Supplementary Table 16: Significant trait associations of LV30 in PhenomeXcan.

Trait description	Sample size	Cases	FDR
Mean reticulocyte volume	344,728		1.09e-32
Mean sphered cell volume	344,729		1.38e-24
Reticulocyte Count	173,480		6.28e-18
Reticulocyte percentage	344,728		1.27e-17
Mean corpuscular haemoglobin concentration	350,468		1.62e-17
Reticulocyte count	344,729		1.62e-17
High light scatter reticulocyte count	344,729		4.78e-11
High Light Scatter Reticulocyte Count	173,480		8.49e-11
Immature reticulocyte fraction	344,728		4.31e-10
High light scatter reticulocyte percentage	344,729		1.21e-05
Mean corpuscular volume	350,473		2.28e-05
Red blood cell (erythrocyte) distribution width	350,473		3.00e-05
Mean platelet (thrombocyte) volume	350,470		6.75e-04
Mean corpuscular haemoglobin	350,472		3.90e-03
Illnesses of adopted mother: Chronic bronchitis/emphysema	2,938	238	1.92e-02

Supplementary Table 17: Significant trait associations of LV30 in eMERGE.

Phecode	Trait description	Sample size	Cases	FDR
No significant associations				

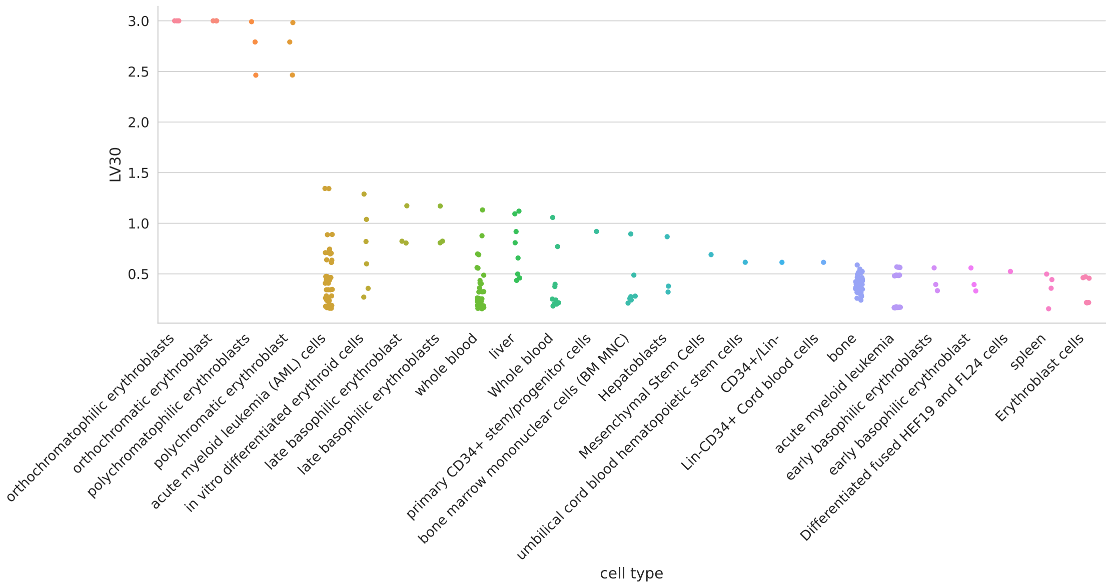

Supplementary Figure 18: **Cell types for LV30.**

LV730

Supplementary Table 18: Pathways aligned to LV730 from the MultiPLIER models.

Pathway	AUC	FDR
DMAP MEGA2	0.82	2.64e-05

Supplementary Table 19: Significant trait associations of LV730 in PhenomeXcan.

Trait description	Sample size	Cases	FDR
Platelet distribution width	350,470		1.13e-10
Mean platelet (thrombocyte) volume	350,470		3.47e-04
Reason former drinker stopped drinking alcohol: Financial reasons	12,110	233	3.71e-02

Supplementary Table 20: Significant trait associations of LV730 in eMERGE.

Phecode	Trait description	Sample size	Cases	FDR
No significant associations				

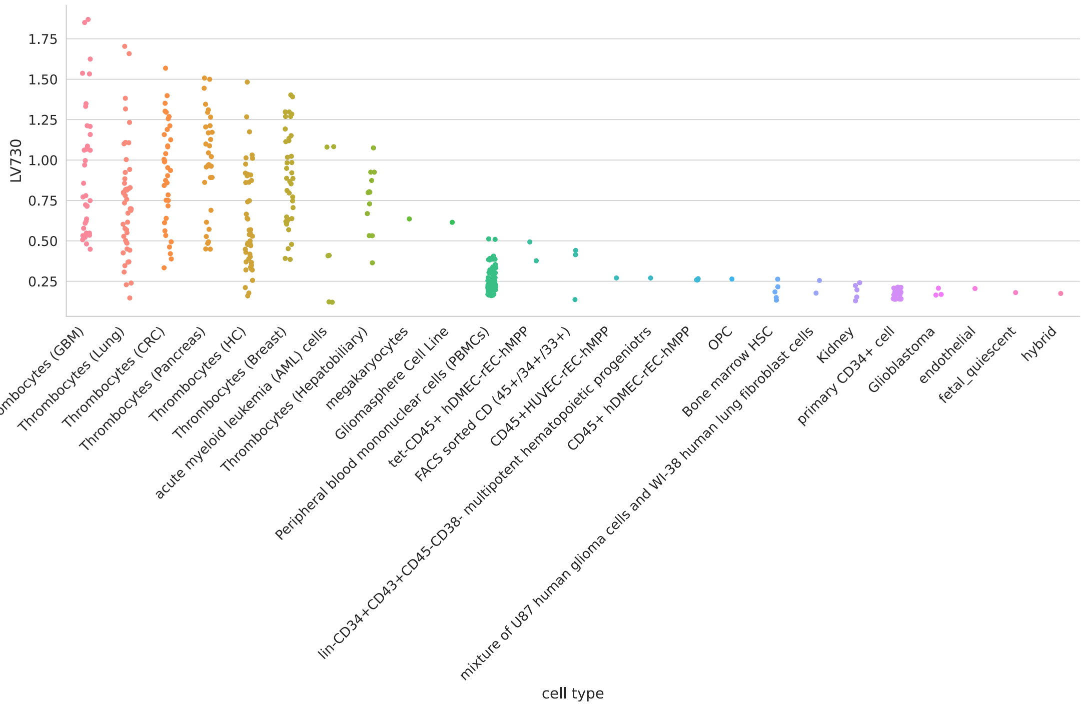

Supplementary Figure 19: **Cell types for LV730.**

LV598

Supplementary Table 21: Pathways aligned to LV598 from the MultiPLIER models.

Pathway	AUC	FDR
PID SYNDECAN 1 PATHWAY	0.81	1.20e-02
REACTOME COLLAGEN FORMATION	0.77	1.89e-02

Supplementary Table 22: Significant trait associations of LV598 in PhenomeXcan.

Trait description	Sample size	Cases	FDR
Corneal resistance factor (right)	76,630		4.05e-04
Corneal resistance factor (left)	76,510		1.86e-03
6mm strong meridian (left)	65,551		2.58e-03
Corneal hysteresis (right)	76,630		1.21e-02
6mm strong meridian (right)	66,256		2.18e-02
Treatment/medication code: evening primrose oil product	361,141	814	2.58e-02
6mm weak meridian (left)	65,551		3.67e-02
Hand grip strength (left)	359,704		4.15e-02
3mm strong meridian (left)	75,398		4.74e-02

Supplementary Table 23: Significant trait associations of LV598 in eMERGE.

Phecode	Trait description	Sample size	Cases	FDR
No significant associations				

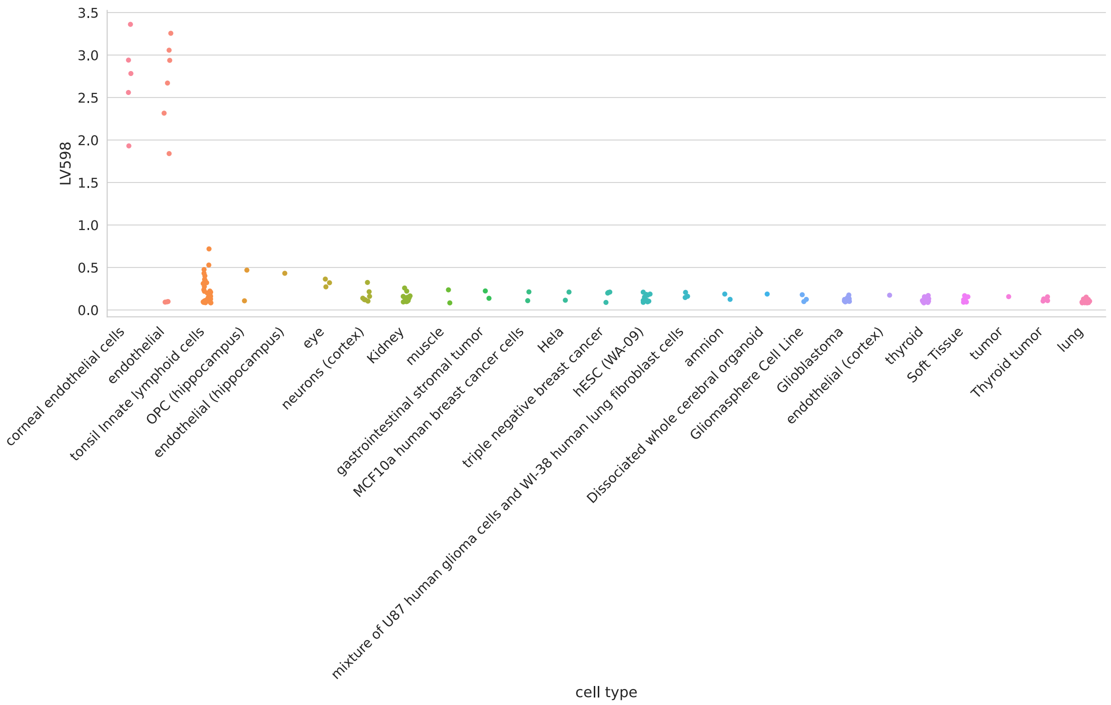

Supplementary Figure 20: **Cell types for LV598.**

LV57

Supplementary Table 24: Pathways aligned to LV57 from the MultiPLIER models.

Pathway	AUC	FDR
KEGG T CELL RECEPTOR SIGNALING PATHWAY	0.70	1.26e-03
SVM T cells CD4 memory activated	0.79	2.59e-03
IRIS CD4Tcell-Th2-restimulated12hour	0.78	7.57e-03
KEGG ALLOGRAFT REJECTION	1.00	1.09e-02
Custom Treg	0.98	1.37e-02
PID NFAT TFPATHWAY	0.74	1.52e-02
IRIS MemoryTcell-RO-activated	0.70	2.87e-02

Supplementary Table 25: Significant trait associations of LV57 in PhenomeXcan.

Trait description	Sample size	Cases	FDR
Non-cancer illness code, self-reported: deep venous thrombosis (dvt)	361,141	7,237	1.76e-13
Blood clot, DVT, bronchitis, emphysema, asthma, rhinitis, eczema, allergy diagnosed by doctor: Blood clot in the leg (DVT)	360,527	7,386	1.22e-12
Diagnoses - main ICD10: I80 Phlebitis and thrombophlebitis	361,194	2,289	7.62e-12
DVT of lower extremities	361,194	2,116	1.27e-09
Venous thromboembolism	361,194	4,620	2.28e-08
DVT of lower extremities and pulmonary embolism	361,194	4,319	4.36e-08
Inflammatory Bowel Disease	34,652	12,882	1.95e-05
hypothyroidism (self-reported)	361,141	17,574	3.84e-05
Medication: levothyroxine sodium	361,141	14,689	8.43e-05
Mouth/teeth dental problems: Mouth ulcers	359,841	36,831	1.02e-03
Crohns Disease	20,833	5,956	1.02e-02
Facial ageing	330,409		1.04e-02
Ulcerative Colitis	27,432	6,968	1.27e-02
Hair colour (natural, before greying): Black	360,270	15,809	1.99e-02
Hair colour (natural, before greying): Light brown	360,270	147,560	4.69e-02

Supplementary Table 26: Significant trait associations of LV57 in eMERGE.

Phecode	Trait description	Sample size	Cases	FDR
286	Coagulation defects	50,182	2,976	1.33e-11
452	Other venous embolism and thrombosis	40,476	3,816	1.52e-05
452.2	Deep vein thrombosis [DVT]	38,791	2,131	4.47e-05
244.4	Hypothyroidism NOS	53,968	9,284	1.12e-02
244	Hypothyroidism	54,404	9,720	1.42e-02

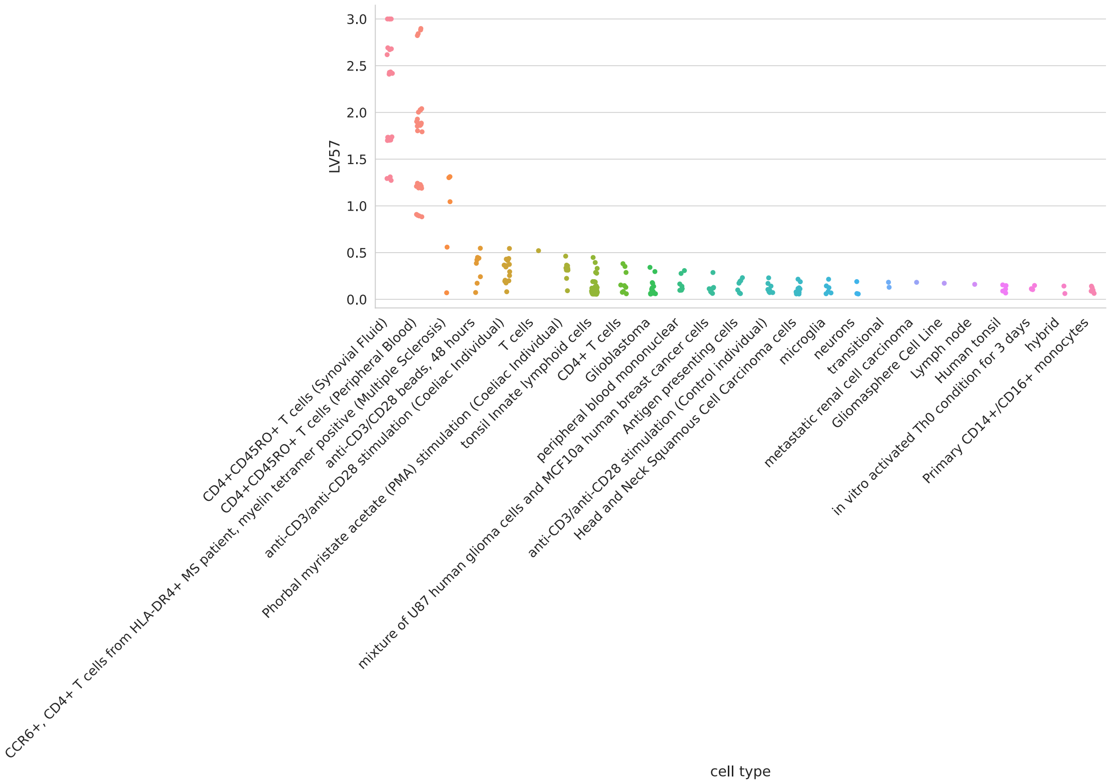

Supplementary Figure 21: **Cell types for LV57.**

LV844

Supplementary Table 27: Pathways aligned to LV844 from the MultiPLIER models.

Pathway	AUC	FDR
KEGG ANTIGEN PROCESSING AND PRESENTATION	0.80	1.35e-03

Supplementary Table 28: Significant trait associations of LV844 in PhenomeXcan.

Trait description	Sample size	Cases	FDR
Non-cancer illness code, self-reported: polymyalgia rheumatica	361,141	753	5.22e-06
Non-cancer illness code, self-reported: type 1 diabetes	361,141	318	4.71e-05
Type 1 diabetes with ketoacidosis	361,194	168	1.03e-04
Age diabetes diagnosed	16,166		3.86e-04
Milk type used: Other type of milk	360,806	4,213	5.48e-04
Non-cancer illness code, self-reported: appendicitis	361,141	3,058	6.12e-04
Diabetic ketoacidosis	361,194	234	7.13e-04
Rheumatoid Arthritis	80,799	19,234	7.46e-04
Type 1 diabetes without complications	361,194	247	1.05e-03
Started insulin within one year diagnosis of diabetes	16,415	1,999	1.30e-03
Insulin medication (males)	165,340	2,248	3.61e-03
Medication: insulin product	361,141	3,545	5.48e-03
Insulin medication (females)	193,148	1,476	7.93e-03
Type 1 diabetes	361,194	583	1.04e-02
Diagnoses - main ICD10: E10 Insulin-dependent diabetes mellitus	361,194	470	1.08e-02
Treatment/medication code: sulfasalazine	361,141	710	1.10e-02
malabsorption/coeliac disease (self-reported)	361,141	1,587	3.12e-02
Job coding: school inspector, education inspector	89,866	238	3.71e-02
Seropositive rheumatoid arthritis	361,194	327	3.86e-02
Non-cancer illness code, self-reported: rheumatoid arthritis	361,141	4,017	4.21e-02
Age hayfever or allergic rhinitis diagnosed by doctor	20,904		4.44e-02
Other/unspecified seropositiverheumatoid arthritis	361,194	299	4.88e-02

Supplementary Table 29: Significant trait associations of LV844 in eMERGE.

Phecode	Trait description	Sample size	Cases	FDR
No significant associations				

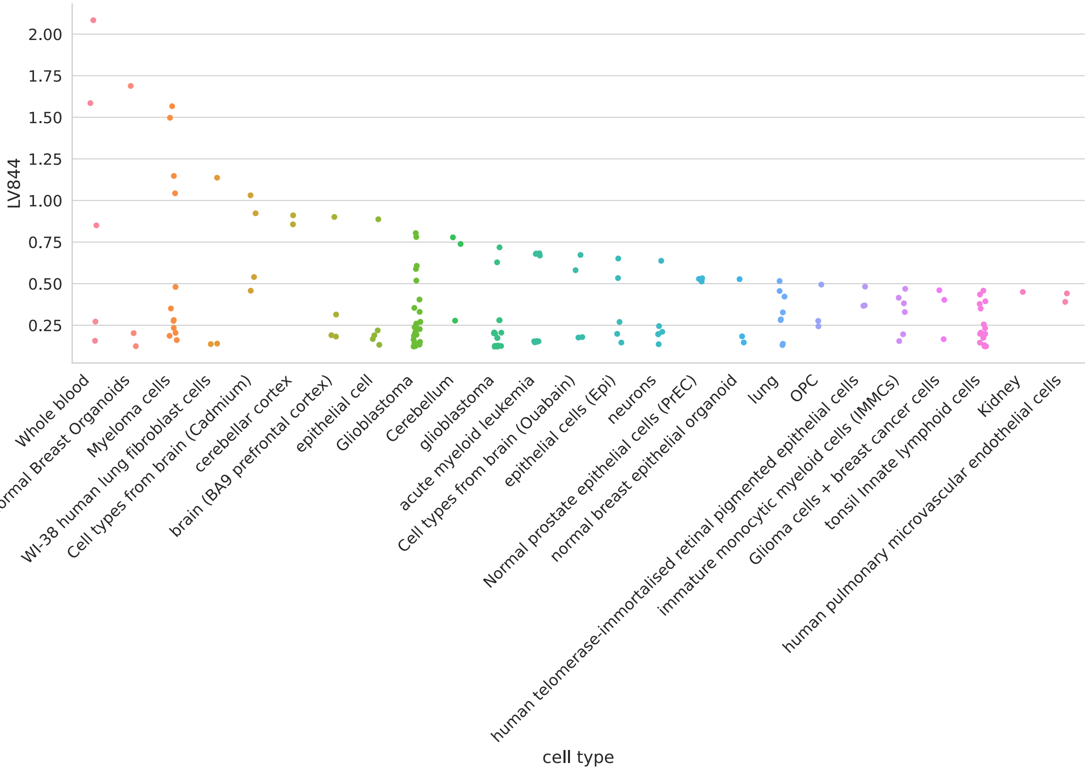

Supplementary Figure 22: **Cell types for LV844.**

LV136

Supplementary Table 30: Pathways aligned to LV136 from the MultiPLIER models.

Pathway	AUC	FDR
PID INTEGRIN1 PATHWAY	0.88	9.35e-06
KEGG ECM RECEPTOR INTERACTION	0.80	7.29e-05
REACTOME COLLAGEN FORMATION	0.87	2.00e-04
REACTOME MUSCLE CONTRACTION	0.75	1.49e-02

Supplementary Table 31: Significant trait associations of LV136 in PhenomeXcan.

Trait description	Sample size	Cases	FDR
Coronary atherosclerosis	361,194	14,334	1.84e-09
Chronic ischaemic heart disease (ICD10 I25)	361,194	12,769	3.52e-09
Ischaemic heart disease (wide definition)	361,194	20,857	3.95e-08
Coronary Artery Disease	184,305	60,801	4.18e-08
3mm strong meridian (right)	75,410		5.54e-05
6mm strong meridian (left)	65,551		1.35e-04
Corneal resistance factor (right)	76,630		2.02e-04
6mm strong meridian (right)	66,256		2.58e-04
Heart attack	360,420	8,288	3.75e-04
Myocardial infarction	361,194	7,018	4.85e-04
Myocardial infarction, strict	361,194	7,018	4.85e-04
3mm strong meridian (left)	75,398		6.65e-04
heart attack/myocardial infarction (self-reported)	361,141	8,239	1.07e-03
6mm weak meridian (left)	65,551		1.10e-03
6mm weak meridian (right)	66,256		1.61e-03
Acute myocardial infarction (ICD10 I21)	361,194	5,948	2.24e-03
3mm weak meridian (right)	75,410		3.69e-03
3mm weak meridian (left)	75,398		3.96e-03
Intra-ocular pressure, Goldmann-correlated (right)	76,630		8.64e-03
6mm asymmetry angle (right)	41,390		1.03e-02
Corneal resistance factor (left)	76,510		1.03e-02
Other specified disorders of muscle	361,194	257	1.09e-02
Major coronary heart disease event excluding revascularizations	361,194	10,157	2.44e-02
Major coronary heart disease event	361,194	10,157	2.44e-02
Non-cancer illness code, self-reported: angina	361,141	11,370	4.53e-02
Eye problems/disorders: Glaucoma	117,890	5,092	4.94e-02

Supplementary Table 32: Significant trait associations of LV136 in eMERGE.

Phecode	Trait description	Sample size	Cases	FDR
411.4	Coronary atherosclerosis	52,836	13,715	1.42e-03

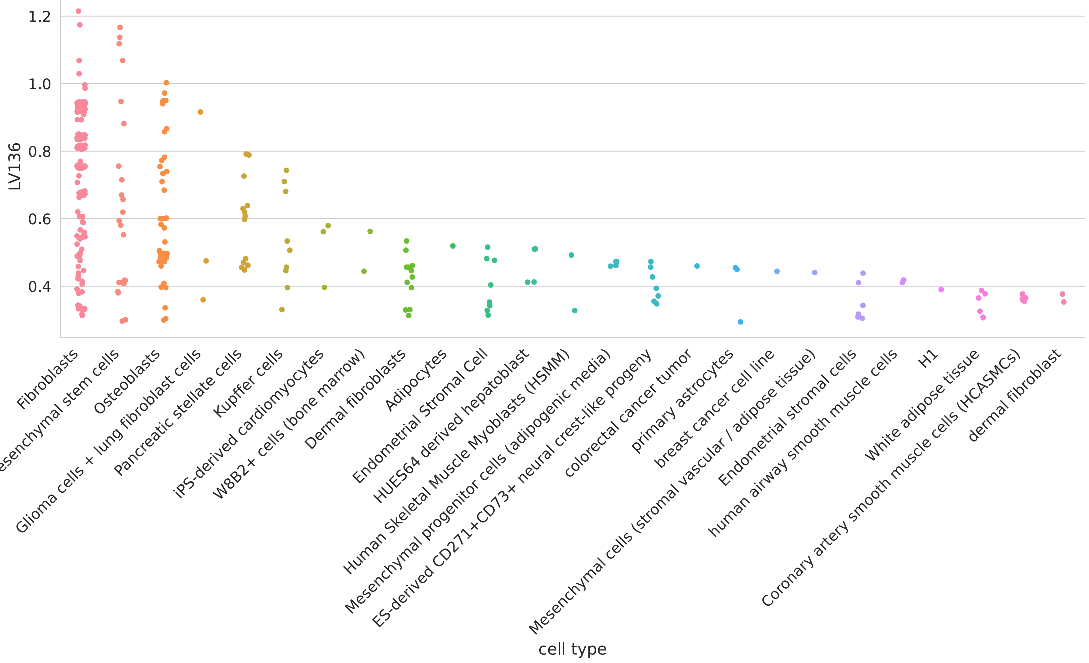

Supplementary Figure 23: **Cell types for LV136.** Pulmonary microvascular endothelial cells were exposed to hypoxia for 24 hours or more[4];

LV21

Supplementary Table 33: Pathways aligned to LV21 from the MultiPLIER models.

Pathway	AUC	FDR
No pathways significantly enriched		

Supplementary Table 34: Significant trait associations of LV21 in PhenomeXcan.

Trait description	Sample size	Cases	FDR
LDL Cholesterol NMR	13,527		1.08e-12
HDL Cholesterol NMR	19,270		3.03e-11
Alzheimers Disease	54,162	17,008	1.96e-09
Triglycerides NMR	21,559		2.05e-09
Illnesses of mother: Alzheimer's disease/dementia	331,041	28,507	1.36e-08
Illnesses of father: Alzheimer's disease/dementia	312,666	15,022	3.15e-08
Illnesses of siblings: Alzheimer's disease/dementia	279,062	1,609	1.55e-07
Any dementia	361,194	243	5.63e-07
Treatment/medication code: simvastatin	361,141	40,921	2.88e-06
Cholesterol lowering medication	193,148	24,247	4.45e-05
High cholesterol (self-reported)	361,141	43,957	9.90e-05
Cholesterol lowering medication	165,340	38,057	4.86e-04
Dementia	361,194	157	9.80e-04
Alzheimer's disease	361,194	119	1.33e-03
Mean reticulocyte volume	344,728		1.76e-03
Father's age at death	266,231		6.68e-03
Illnesses of mother: None of the above (group 1)	332,611	138,291	1.42e-02
ECG, phase time	53,998		1.60e-02
Treatment/medication code: atorvastatin	361,141	10,805	2.92e-02
Mean sphered cell volume	344,729		3.33e-02
Non-cancer illness code, self-reported: cellulitis	361,141	232	3.40e-02
Medication for cholesterol, blood pressure or diabetes (males)	165,340	110,372	3.66e-02
Mother still alive	355,029	140,246	4.96e-02

Supplementary Table 35: Significant trait associations of LV21 in eMERGE.

Phecode	Trait description	Sample size	Cases	FDR
272.1	Hyperlipidemia	55,843	29,195	4.22e-03
272	Disorders of lipoid metabolism	55,892	29,244	4.50e-03

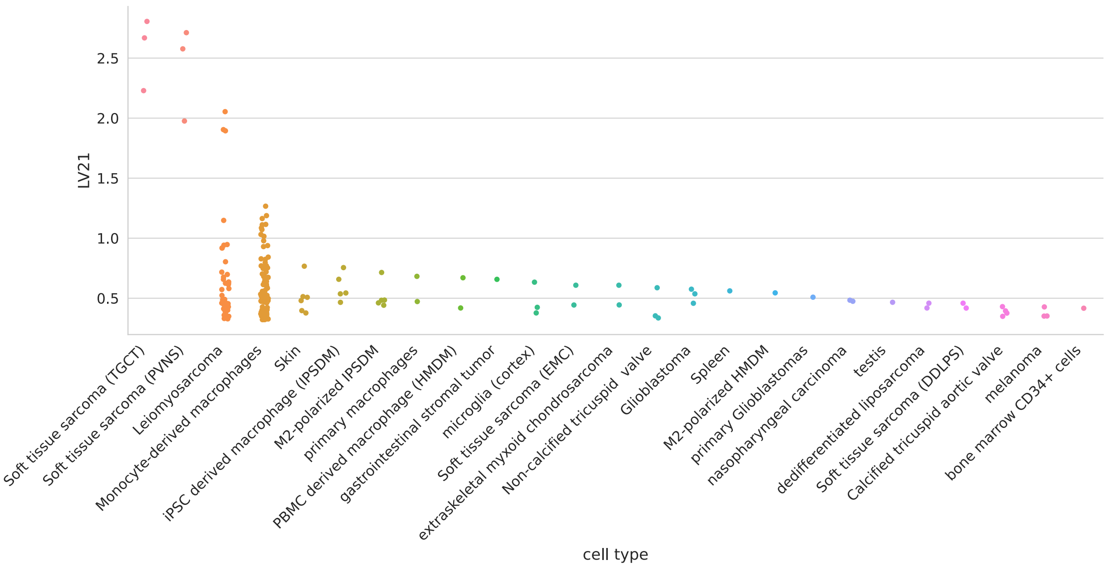

Supplementary Figure 24: **Cell types for LV21.**

Supplementary References

1. Barbeira, A. N. *et al.* Fine-mapping and QTL tissue-sharing information improves the reliability of causal gene identification. *Genetic Epidemiology* **44**, 854–867 (2020).
2. *Harmonization and Imputation Overview.* (hakyimlab, 2022).
3. Korotkevich, G. *et al.* Fast gene set enrichment analysis. (2016) doi:10.1101/060012.
4. Homo sapiens (ID 232177) - BioProject - NCBI. https://www.ncbi.nlm.nih.gov/bioproject/PRJNA232177.
