# Supplementary material for: Projecting genetic associations through gene expression patterns highlights disease etiology and drug mechanisms
Source: Nat Commun. 2023 Sep 9;14:5562. doi: 10.1038/s41467-023-41057-4 (PMC10492839; doi:10.1038/s41467-023-41057-4)
Supplement: Supplementary file 3 — Description of Additional Supplementary Files [file 41467_2023_41057_MOESM3_ESM.pdf]

File name: Supplementary Data 1
Description: List of traits in PhenomeXcan, with information about trait code, description, type (binary, categorical, continuous, ordinal), sample size, number of cases, number of controls, and study source.

File name: Supplementary Data 2
Description: Archive containing two files about the CRISPR-Cas9 screening on lipid regulation: 1) a list of 462 differentially expressed genes, their effect on lipids (decrease or increase), and rank, and 2) information about the ranking criteria for genes.

File name: Supplementary Data 3
Description: Clustering partition with *k*=16 groups of 3,752 traits from PhenomeXcan projected into the latent space (with 987 latent variables).

File name: Supplementary Data 4
Description: Clustering partition with *k*=22 groups of 3,752 traits from PhenomeXcan projected into the latent space (with 987 latent variables).

File name: Supplementary Data 5
Description: Clustering partition with *k*=25 groups of 3,752 traits from PhenomeXcan projected into the latent space (with 987 latent variables).

File name: Supplementary Data 6
Description: Clustering partition with *k*=26 groups of 3,752 traits from PhenomeXcan projected into the latent space (with 987 latent variables).

File name: Supplementary Data 7
Description: Clustering partition with *k*=29 groups of 3,752 traits from PhenomeXcan projected into the latent space (with 987 latent variables).

File name: Supplementary Data 8
Description: Readable format of primer sequences for generating Illumina libraries.
