# Supplementary material for: Projecting genetic associations through gene expression patterns highlights disease etiology and drug mechanisms
Source: Nat Commun. 2023 Sep 9;14:5562. doi: 10.1038/s41467-023-41057-4 (PMC10492839; doi:10.1038/s41467-023-41057-4)
Supplement: Supplementary file 13 — Reporting Summary [file 41467_2023_41057_MOESM13_ESM.pdf]

nature portfolio

Corresponding author(s): Casey Greene, PhD

Last updated by author(s): Jun 26, 2023

Reporting Summary

Nature Portfolio wishes to improve the reproducibility of the work that we publish. This form provides structure for consistency and transparency in reporting. For further information on Nature Portfolio policies, see our Editorial Policies and the Editorial Policy Checklist.

Statistics

For all statistical analyses, confirm that the following items are present in the figure legend, table legend, main text, or Methods section.

n/a	Confirmed	
☐	☒	The exact sample size (*n*) for each experimental group/condition, given as a discrete number and unit of measurement
☐	☒	A statement on whether measurements were taken from distinct samples or whether the same sample was measured repeatedly
☐	☒	The statistical test(s) used AND whether they are one- or two-sided<br>*Only common tests should be described solely by name; describe more complex techniques in the Methods section.*
☐	☒	A description of all covariates tested
☐	☒	A description of any assumptions or corrections, such as tests of normality and adjustment for multiple comparisons
☐	☒	A full description of the statistical parameters including central tendency (e.g. means) or other basic estimates (e.g. regression coefficient) AND variation (e.g. standard deviation) or associated estimates of uncertainty (e.g. confidence intervals)
☐	☒	For null hypothesis testing, the test statistic (e.g. *F*, *t*, *r*) with confidence intervals, effect sizes, degrees of freedom and *P* value noted<br>*Give P values as exact values whenever suitable.*
☒	☐	For Bayesian analysis, information on the choice of priors and Markov chain Monte Carlo settings
☒	☐	For hierarchical and complex designs, identification of the appropriate level for tests and full reporting of outcomes
☐	☒	Estimates of effect sizes (e.g. Cohen's *d*, Pearson's *r*), indicating how they were calculated

Our web collection on statistics for biologists contains articles on many of the points above.

Software and code

Policy information about availability of computer code

Data collection	We used FlowJo v10.7 and FACS Jazz Software v1.1
Data analysis	The code necessary to reproduce all the analyses in this work is available at https://doi.org/10.5281/zenodo.8044955 and the GitHub repository https://github.com/greenelab/phenoplier.<br><br>We used Python 3.8 and R 3.6 with several computational packages. The main Python packages used were: Jupyter Lab (2.2), pandas (1.1), matplotlib (3.3), seaborn (0.11), numpy (1.19), scipy (1.5), scikit-learn (0.23), and umap-learn (0.4). The main R packages were: Bioconductor (3.10), clusterProfiler (3.14), clustree (0.4), and fgsea (1.17). We also developed several scripts and notebooks which are published under an open-source license. We documented all the steps necessary to carry out all the analyses. We also provide a Docker image to use the same runtime environment we used, and a demo to quickly test the methods on real data.

For manuscripts utilizing custom algorithms or software that are central to the research but not yet described in published literature, software must be made available to editors and reviewers. We strongly encourage code deposition in a community repository (e.g. GitHub). See the Nature Portfolio guidelines for submitting code & software for further information.

Data

Policy information about availability of data

All manuscripts must include a data availability statement. This statement should provide the following information, where applicable:

- Accession codes, unique identifiers, or web links for publicly available datasets
- A description of any restrictions on data availability
- For clinical datasets or third party data, please ensure that the statement adheres to our policy

All the main datasets generated in this study are available at https://doi.org/10.5281/zenodo.8044955 and the GitHub repository https://github.com/greenelab/phenoplier.

The main input datasets used are TWAS from PhenomeXcan (https://doi.org/10.1126/sciadv.aba2083) for 4,091 traits and from the Electronic Medical Records and Genomics (eMERGE) network phase III (https://doi.org/10.1101/2021.10.21.21265225) for 309 traits; transcriptional responses to small molecule perturbations from LINCS L1000 (https://doi.org/10.1016/j.cell.2017.10.049) that were further preprocessed and mapped to DrugBank IDs from (https://doi.org/10.5281/zenodo.47223); latent space/gene module models from MultiPLIER (https://doi.org/10.1016/j.cels.2019.04.003).

The data used from PhenomeXcan, LINCS L1000, and MultiPLIER are publicly available. All significant results reported for the eMERGE and Penn Medicine BioBank (PMBB) phenome-wide TWAS are contained in (https://doi.org/10.1101/2021.10.21.21265225). The individual-level PMBB raw datasets can not be made publicly available due to institutional privacy policy. Please contact Penn Medicine Biobank (https://pmbb.med.upenn.edu/pmbb/) for requests of access to data. eMERGE network phase III data is available on dbGAP (Accession: phs001584.v2.p2).

Human research participants

Policy information about studies involving human research participants and Sex and Gender in Research.

Reporting on sex and gender	N/A
Population characteristics	N/A
Recruitment	N/A
Ethics oversight	N/A

Note that full information on the approval of the study protocol must also be provided in the manuscript.

Field-specific reporting

Please select the one below that is the best fit for your research. If you are not sure, read the appropriate sections before making your selection.

☒ Life sciences ☐ Behavioural & social sciences ☐ Ecological, evolutionary & environmental sciences

For a reference copy of the document with all sections, see nature.com/documents/nr-reporting-summary-flat.pdf

Life sciences study design

All studies must disclose on these points even when the disclosure is negative.

Sample size	GWAS sample sizes across all 4,091 traits are provided in the PhenomeXcan study (https://doi.org/10.1126/sciadv.aba2083) and eMERGE for 309 traits (https://doi.org/10.1101/2022.02.22.22271350). The RNA-seq data from recount2 (https://doi.org/10.1038/nbt.3838) and preprocessed in the MultiPLIER study (https://doi.org/10.1016/j.cels.2019.04.003) contains 6750 genes measured across 37032 samples. All these datasets were obtained from external studies, and sample sizes were determined in those.
Data exclusions	Data were not excluded from the analyses.
Replication	To replicate our findings in PhenomeXcan, we used different approaches. Gene module-trait associations were replicated by using 1) genes detected in a CRISPR screen to analyze lipid accumulation, 2) modules with known trait associations that were previously analyzed in a different study with independent datasets (MultiPLIER, https://doi.org/10.1016/j.cels.2019.04.003), 3) TWAS results from eMERGE (an independent cohort).
Randomization	We generated 1000 random phenotypes to verify that p-values from the regression model were callibrated. For the clustering pipeline, we simulated two escenarios where there is no structure in the input data matrix (gene-trait associations from PhenomeXcan projected into the latent gene expression representation): 1) the gene-trait association matrix does not have any meaningful structure to find groups of traits (by randomly shuffling genes for each trait), while preserving the latent variables from the MultiPLIER models; and 2) the latent variables do not have any meaningful structure to find groups of traits (by randomly shuffling latent variables in the projected data), while preserving the gene-trait association matrix.

Blinding	This is not relevant in the study. We did not perform any clinical trials. We performed simulations for computational methods applied to existing genetic and transcriptomic data.

Reporting for specific materials, systems and methods

We require information from authors about some types of materials, experimental systems and methods used in many studies. Here, indicate whether each material, system or method listed is relevant to your study. If you are not sure if a list item applies to your research, read the appropriate section before selecting a response.

Materials & experimental systems

n/a	Involved in the study	
☒	☐	Antibodies
☐	☒	Eukaryotic cell lines
☒	☐	Palaeontology and archaeology
☒	☐	Animals and other organisms
☒	☐	Clinical data
☒	☐	Dual use research of concern

Methods

n/a	Involved in the study	
☒	☐	ChIP-seq
☐	☒	Flow cytometry
☒	☐	MRI-based neuroimaging

Eukaryotic cell lines

Policy information about cell lines and Sex and Gender in Research

Cell line source(s)	Hep G2 [HEPG2] is a cell line exhibiting epithelial-like morphology that was isolated from a hepatocellular carcinoma of a 15 year-old, White, male youth with liver cancer.
Authentication	HepG2 cell line was purchase from ATCC (ATCC® HB-8065™) and was authenticated by ATCC. The cell line was authenticated by human STR profiling.
Mycoplasma contamination	Cell lines were not tested for mycoplasma contamination. The cell line was authenticated by human STR profiling.
Commonly misidentified lines (See ICLAC register)	None

Flow Cytometry

Plots

Confirm that:

☒ The axis labels state the marker and fluorochrome used (e.g. CD4-FITC).

☒ The axis scales are clearly visible. Include numbers along axes only for bottom left plot of group (a 'group' is an analysis of identical markers).

☒ All plots are contour plots with outliers or pseudocolor plots.

☒ A numerical value for number of cells or percentage (with statistics) is provided.

Methodology

Sample preparation	Crispr gene screen pool was added at an MOI between 0.3-0.4 to ensure 95% of infected HepG2 cells get only one viral particle per cell, ~200M HepG2 cells were initiated for the screen. Transduction was carried out in the similar fashion as de-scribed above. Briefly, 2.5M cells were seeded in each well of 14 6-well plates, along with 8ug/ml of polybrene. Volume of 120ul of virus was added to each experimental well. 18hrs post transduction, virus/PB mix medium was removed, and cells in each well were collect-ed, counted, and pooled into T175 flasks. At 60hr post transduction, 2ug/ml of puromycin was added to each flask. Mediums were changed every 2 days with fresh EMEM, topped with 2ug/ml puromycin. 7 days after puromycin selection, cells were collected, pooled, counted, and replated. 9 days after puromycin selection, cells were assigned to 2 groups. 20-30M cells were collected as Unsorted Control. Cell pellet was spun down at 500 x g for 5min at 4oC. Dry pellet was kept at -80oC for further genomic DNA isolation. The rest of the cells (approximately 200M) were kept in 100mm dishes, and stained with fluo-rescent dye (LipidSpotTM 488, Biotium, Cat. 70065-T). In Brief, LipidSpot 488 was diluted to 1:100 with DPBS. 4ml of staining solution was used for each dish, and incubated at 37oC for 30min.
Instrument	FACSJazz cell sorter.
Software	We used FlowJo software package for analysis of all the flow data and FACS Jazz Software for all sorting
Cell population abundance	HepG2 lentivirus pool treated cells were flow sorted into 2 groups GFP- (low fat droplet staining) and GFP+ (high fat droplet staining) cells. Cell abundance from 20,000 cell sorts for GFP- were ~3,700 cells (18.7% of total cells sorted) and GFP+ were

~3,400 cells (17.1% of total cells sorted). A total of 200 million cells were flow sorted.

Gating strategy

The gating strategy was to gate all single cells (Singlets) to exclude any cells that are clumped. All cells found in the singlet gate were then gated to exclude dead cells (Cells). The final gates, GFP- (SSC 56, FLA-1 10^1) and GFP+ (SSC 56, FLA-1 10^2) were gated so each population could be sorted into separate tubes.

☐ Tick this box to confirm that a figure exemplifying the gating strategy is provided in the Supplementary Information.
